# Supplementary material for: EnzML: multi-label prediction of enzyme classes using InterPro signatures
Source: BMC Bioinformatics. 2012 Apr 25;13:61. doi: 10.1186/1471-2105-13-61 (PMC3483700; doi:10.1186/1471-2105-13-61)
Supplement: Addtional file 5 — The Java code to format the data files, evaluate and predict. The file enzml_java_code.tar.gz contains the Java code used to format database data to ARFF and XML formats, to execute cross and train-test (jackknife) evaluations and to record evaluation results to database. More information is included in the readme.txt file and the Javadoc files. The code can be used with a MySQL database. To use a different database software, other JDBC drivers might be required. [file 1471-2105-13-61-S5.gz › java_code/enzml2011/doc/uk/ac/ed/inf/taweka/Initialisable.html]

Initialisable


---


|  |  |  |
| --- | --- | --- |
| **Overview** \| **Package** \| **Class** \| **Use** \| **Tree** \| **Deprecated** \| **Index** \| **Help** | |  |
| PREV CLASS   **NEXT CLASS** | **FRAMES**    **NO FRAMES**     **All Classes** |  |
| SUMMARY: NESTED \| FIELD \| CONSTR \| METHOD | DETAIL: FIELD \| CONSTR \| METHOD |  |


---


## uk.ac.ed.inf.taweka Interface Initialisable

**All Known Implementing Classes:**: Arff, ArffProperties, AttributesFilter, AttributesFilteredArff, Initialised, MulanArff, MulanArffProperties, MulanArffRecord, MulanCrossExperimenter, MulanPredict, MulanPredictWithTrainedModel, TrainTestExperimenter, TrainTestExperimenterSerialized

---

``` public interface Initialisable ```

A class having a boolean variable telling whether the initialisation has
completed correctly

**Version:**
:   29 Sep 2010

**Author:**
:   Luna De Ferrari luna.deferrari-at-ed.ac.uk

---

| **Method Summary** | |
| --- | --- |
| `boolean` | `initialisationIsCorrect()` |
| `void` | `setInitialisationIsCorrect(boolean isCorrect)` |

**Method Detail**

### initialisationIsCorrect

```
boolean initialisationIsCorrect()
```

---


### setInitialisationIsCorrect

```
void setInitialisationIsCorrect(boolean isCorrect)
```


---


|  |  |  |
| --- | --- | --- |
| **Overview** \| **Package** \| **Class** \| **Use** \| **Tree** \| **Deprecated** \| **Index** \| **Help** | |  |
| PREV CLASS   **NEXT CLASS** | **FRAMES**    **NO FRAMES**     **All Classes** |  |
| SUMMARY: NESTED \| FIELD \| CONSTR \| METHOD | DETAIL: FIELD \| CONSTR \| METHOD |  |


---
